# Longitudinal evaluation of health-related quality of life in Chinese patients with early-stage HER2-positive breast cancer undergoing neratinib extended adjuvant therapy: a real-world prospective cohort study

**DOI:** 10.3389/fonc.2026.1828599

**Published:** 2026-08-12

**Authors:** Xuanhua Liang, Zibai Guo, Jinhong Wei, Anping Gui, Mengru Jian, Jie Min, Mengwen Wang, Shihui Ma

**Affiliations:** Breast Center Zone One, Zhongshan City People’s Hospital, Zhongshan, Guangdong, China

**Keywords:** diarrhea, HER2-positive breast cancer, neratinib, patient-reported outcomes, quality of life

## Abstract

**Background:**

Neratinib, an irreversible pan-HER tyrosine kinase inhibitor, is a well-established extended adjuvant therapy for early-stage human epidermal growth factor receptor 2-positive (HER2+) breast cancer. However, its clinical application is often constrained by the high incidence of treatment-related diarrhea. This study sought to assess the dynamic changes in health-related quality of life (HRQoL) and the tolerability of neratinib, with a particular focus on dose escalation strategies and anti-diarrheal prophylaxis, within a real-world Chinese patient cohort.

**Methods:**

This single-center, prospective observational study included 31 female patients diagnosed with stage I-III HER2-positive breast cancer who initiated neratinib therapy following the completion of trastuzumab-based adjuvant treatment between October 2022 and January 2024. Patients were administered either non–dose-escalation group (240 mg/day) or dose-escalation group (120 mg/160 mg daily during Week 1, 160 mg/200 mg daily during Week 2, and 240 mg daily from Week 3 onward). Diarrhea management was conducted using prophylactic or on-demand loperamide. HRQoL was evaluated longitudinally through the EQ-5D-5L (health utility index and EQ visual analog scale [EQ VAS]) and Functional Assessment of Chronic Illness Therapy-Diarrhea (FACIT-D) questionnaires at predefined time points: baseline (D1), D30, D60, D90, D180, and D360. Diarrhea severity was graded according to Common Terminology Criteria for Adverse Events v5.0 (CTCAE v5.0) criteria. Statistical analyses encompassed descriptive statistics and Wilcoxon signed-rank tests.

**Results:**

The median age of the patients was 48 years (range: 37–65 years), with 58.1% and 41.9% classified as pathological stage II and III, respectively. A total of 64 diarrhea events were recorded (grade 1, 48.4%; grade 2, 50.0%; grade 3, 1.6%), with no grade 4 events or fatalities. Permanent discontinuation of treatment due to diarrhea occurred in 16.1% of patients, 23.5% in the dose-escalation group vs. 7.1% in the non-dose-escalation group. HRQoL temporarily declined during the early treatment phase, as evidenced by a reduction in the EQ-5D-5L health utility index from 0.96 ± 0.07 at baseline to 0.89 ± 0.15 at D30 (p=0.011) and a decrease in the EQ VAS score from 89.94 ± 9.92 to 84.16 ± 12.64 at D30 (p=0.018). However, HRQoL demonstrated progressive recovery from D90 onward, with scores surpassing baseline levels by D180 (EQ-5D-5L utility index: 0.96 ± 0.07; EQ VAS: 89.92 ± 7.74) and D360 (utility index: 0.97 ± 0.06; EQ VAS: 92.84 ± 7.19). The FACIT-D score showed an initial improvement (baseline: 58.81 ± 7.95 vs. D30: 66.90 ± 11.39, p=0.002), followed by stabilization at baseline levels from D180 onward. Notably, no disease recurrence or distant metastasis was observed in patients who completed the treatment.

**Conclusions:**

Neratinib-associated HRQoL impairment is transient, with initial declines primarily due to diarrhea, which typically resolves within 3–6 months of treatment. With the implementation of appropriate supportive measures, such as loperamide prophylaxis and dose modifications, the majority of patients experience sustained HRQoL recovery, and even improvement, over a 12-month period.

## 1 Introduction

Breast cancer is one of the most prevalent malignant tumors among women globally, with human epidermal growth factor receptor 2-positive (HER2+) breast cancer accounting for approximately 15%-20% of all cases (1). For patients with early-stage HER2-positive breast cancer, the current standard treatment regimen primarily consists of chemotherapy combined with anti-HER2 targeted therapy. However, in the absence of effective therapeutic interventions, the prognosis for patients with HER2-positive breast cancer is significantly worse compared to those with HER2-negative disease, characterized by markedly higher rates of recurrence and metastasis (2, 3). The ExteNet study (4) demonstrated that neratinib, an irreversible pan-HER tyrosine kinase inhibitor, serves as an extended adjuvant treatment option for patients with HER2-positive early breast cancer, offering substantial clinical benefits (5). Specifically, neratinib increased the 5-year invasive disease-free survival rate to 90.2% in the ExteNet trial. However, the treatment was associated with a high incidence of grade 3 diarrhea, reported in 40% of patients, and 17% of patients discontinued treatment due to this adverse event (6).

Despite the potential therapeutic value of natural products as supportive treatments for breast cancer (7), effective anti-diarrheal strategies remain insufficient, and diarrhea continues to be the most common adverse event associated with neratinib therapy. Furthermore, diarrhea is also among the most common adverse reactions linked to the use of other tyrosine kinase inhibitors (TKIs) (8, 9). The CONTROL trial (10) investigated anti-diarrheal prophylactic measures and neratinib dose escalation strategies, demonstrating that the dose escalation strategy (120 mg → 160 mg → 240 mg, abbreviated as DE strategy) significantly reduced the incidence of grade 3 diarrhea to 13%. However, the evaluation of patient-reported outcomes (PROs) in this trial was relatively limited, utilizing the EQ-5D-5L (11, 12) and FACT-B (13, 14) questionnaires to assess the impact on quality of life (QoL), without conducting a comprehensive analysis or discussion of its dynamic changes. This study aims to validate the applicability of the neratinib dose escalation strategy in Chinese patients through real-world observations, while simultaneously employing longitudinal PRO assessments at multiple pre-specified time points (15, 16).

## 2 Methods

### 2.1 Study design

This study was a single-center, observational, prospective cohort study conducted between October 2022 and January 2024, and was approved by the Ethics Committee of the People’s Hospital of Zhongshan City (approval number: K2022-161). Prior to enrollment, written informed consent was obtained from all participants.

Inclusion criteria:

1. Age ≥18 years, stage I-III HER2-positive breast cancer;
2. Completed adjuvant therapy with trastuzumab for ≤1 year and decided to use neratinib as extended adjuvant treatment.

Exclusion criteria:

1. Metastatic breast cancer;
2. Active intestinal disease.

### 2.2 Outcome

The primary aim of this study was to assess PROs and the dynamic effects of neratinib on patients’ QoL. Secondary objectives encompassed the evaluation of patients’ disease characteristics, prior treatment history, neratinib utilization patterns, and disease prognosis.

The loading dose regimen for neratinib was established at 240 mg/day for a duration of 12 months.

Patients were randomized into two dosing strategy groups (**Supplementary Figure 1**). In the non–dose-escalation group (n=14), participants received a fixed oral dose of neratinib at 240 mg/day. In the dose-escalation group (n=17), participants received neratinib at 120 mg/day or 160 mg/day during Week 1, 160 mg/day or 200 mg/day during Week 2, and 240 mg/day from Week 3 onward, following the dose-escalation strategy implemented in the CONTROL trial (10). All patients were permitted to continue their prescribed oral endocrine therapy as directed by their treating physicians.

In the dose-escalation group, loperamide prophylaxis was initiated in a subset of patients (n=7), with a preventive oral regimen of 4 mg daily administered from Day 1 to Day 14.

All patients in this study, regardless of group assignment, were permitted to use loperamide for the management of diarrhea. All patients were permitted to use additional loperamide as required, with a maximum allowable dose of 16 mg/day.

### 2.3 Data collection

The detailed assessment parameters and corresponding time points are summarized in **Table 1**.

**Table 1:** Assessment parameters, tools/standards, and time points.

Parameters	Tool/Standard	Time Point
Diarrhea Grading	CTCAE v5.0	NA
Health utility value and EQ VAS	EQ-5D-5L Chinese version utility index (17)	D1/D30/D60/D90/D180/D360
Diarrhea-Related Quality of Life	FACIT-D Scale	D1/D30/D60/D90/D180/D360

### 2.4 HRQoL questionnaires

This study utilized the Chinese EQ-5D-5L value set, including its culturally adapted descriptive system and China-specific scoring algorithm developed by Luo et al. (17), which generates utility scores ranging from –0.391 to 1. A score of 1 indicates full health, 0 represents a health state equivalent to death, and negative values denote health states perceived as worse than death. As requested by the copyright holder, the EQ-5D-5L instrument was not supplied or administered in accordance with the EuroQol Research Foundation’s standardized protocol.

The Functional Assessment of Chronic Illness Therapy–Diarrhea (FACIT-D) consists of the Functional Assessment of Cancer Therapy–General (FACT-G) and a symptom-specific subscale designed to assess QOL related to diarrhea (13, 18). The FACIT-D items are rated on 5-point Likert-type scales ranging from 0 to 4, with higher scores reflecting better QOL.

Permission to use the EQ-5D-5L and FACIT-D instruments was obtained from their respective copyright holders. Documentation verifying these permissions is retained by the corresponding author and can be provided upon request.

### 2.5 Statistical analysis

The data cutoff date for this analysis was August 31, 2025. All data collected up to this date were included, and patient follow-up status was assessed at this time point.

Descriptive statistics were employed to evaluate patient demographics, tumor characteristics, and treatment-related variables. The EQ-5D-5L and FACIT-D scores at time points D1, D30, D60, D90, D180, and D360 were summarized using measures of central tendency and dispersion, including mean, median, and standard deviation. The Kolmogorov-Smirnov test was utilized to determine the normality of score distributions. As the score distributions at most time points deviated from normality, and for scores not adhering to the assumption of normality, primary comparisons between baseline (D1) and subsequent time points were conducted using the Wilcoxon signed-rank test.

Given the exploratory design of this study and the limited sample size, no formal adjustments for multiple comparisons (such as Bonferroni correction) were performed. All reported P-values are nominal; therefore, the findings should be interpreted with appropriate caution and considered primarily hypothesis-generating.

All statistical procedures were executed using R statistical software version 27.0.1.0, with statistical significance defined as a two-sided p-value of less than 0.05 (19).

## 3 Results

### 3.1 Baseline characteristics

As of August 2025, this study has enrolled and analyzed a cohort of 31 female patients diagnosed with early-stage breast cancer who underwent neratinib-based intensified adjuvant therapy.

The median age of the patients was 48 years (range: 37–65 years). The majority of patients were classified as pathological stage II (58.1%) and stage III (41.9%), with no cases observed in stage I or IV. Hormone receptor positivity (ER+ and/or PgR+) was identified in 64.5% of the tumors. All patients had previously received trastuzumab, taxanes, and pertuzumab as part of their treatment regimen. Additionally, 18 patients (58.1%) had undergone anthracycline-based therapy (**Table 2**).

**Table 2:** Baseline characteristics of patients (N=31).

Variable	n (%)
Female	31 (100.0%)
Age (years), Median (range)	48 (37-65)
Cancer stage	
II	18 (58.1%)
III	13 (41.9%)
Menopausal status	
premenopausal	6 (19.3%)
postmenopausal	23 (74.2%)
NA	2 (6.5%)
Hormone receptor status	
Positive(ER+ and/or PgR+)	20 (64.5%)
Negative(ER- and PgR-)	11 (35.5%)
Prior radiotherapy	0 (0.0%)
Prior (neo)adjuvant therapy	
Trastuzumab	31 (100.0%)
Taxanes	31 (100.0%)
Anthracycline	18 (58.1%)
Pertuzumab	31 (100.0%)
T-DM1	0 (0.0%)
Comorbidities	
Hypertension	3 (9.7%)
Diabetes	2 (6.5%)
Other tumors	1 (3.2%)
Previous treatment toxicities	
Hematologic	13 (41.9%)
Hepatic	1 (3.2%)
Adjuvant endocrine therapy	19 (61.3%)
SERM only	4 (12.9%)
SERM + OFS	2 (6.5%)
AI only	9 (29.0%)
AI + OFS	4 (12.9%)

ER, estrogen receptor; PgR, progesterone receptor; NA, information not available; AI, aromatase inhibitors; OFS, ovarian function suppression; SERM, selective estrogen receptor modulator.

Regarding the distribution of neratinib dosing, a total of 14 patients (45.2%) initiated treatment at the full dose of 240 mg orally once daily (non−dose−escalation group). The remaining 17 patients (54.8%) were assigned to the dose−escalation group and commenced treatment at reduced starting doses: 2 patients (6.4%) at 120 mg once daily and 15 patients (48.4%) at 160 mg once daily, with subsequent up-titration to 240 mg/day.

In terms of diarrhea prophylaxis, 7 patients (22.6%) received loperamide at the start of treatment for prevention, whereas 24 patients (77.4%) did not receive loperamide.

As of the data cutoff (August 31, 2025), all patients had either completed treatment or prematurely discontinued therapy. Among these, 25 patients (80.6%) completed at least one year of neratinib treatment, while 6 patients (19.4%) discontinued therapy due to adverse events (AEs), with diarrhea being the cause of discontinuation in 5 patients (16.1%).

Regarding treatment duration, the mean duration was 10.3 ± 4.8 months, with a median duration of 12.2 months and an interquartile range (IQR) of 11.6–12.3 months (**Table 3**). Notably, patients who initiated neratinib at the full dose demonstrated longer treatment durations compared to those who started at reduced doses. The highest rates of permanent discontinuation occurred within the first three months of treatment.

**Table 3:** Study disposition and treatment exposure.

Variable	n (%)
Patients (N=31)	
Completed 1 year of treatment	25 (80.6%)
Discontinued treatment prior to 1 year	6 (19.4%)
Permanent discontinued due to any AE	6 (19.4%)
Permanent discontinued due to diarrhea	5 (16.1%)
Treatment duration, months	
Mean (± SD)	10.3 (4.8)
Median	12.2
IQR	11.6-12.3
Initial dose, mg/day	
120mg	2 (6.4%)
160mg	15 (48.4%)
240mg	14 (45.2%)
Antidiarrheal regimen at the start	
Loperamide	7 (22.6%)
Without loperamide	24 (77.4%)

AE, Adverse Event; SD, standard deviation; IQR, interquartile range.

### 3.2 Disease Prognosis

No evidence of disease recurrence or distant metastasis was detected during the follow-up period in patients who completed the full treatment regimen.

### 3.3 Diarrhea safety analysis

During the observation period, a total of 64 diarrhea events were recorded (**Table 4**). Among these, 31 (48.4%) were Grade 1, 32 (50.0%) were Grade 2, and 1 (1.6%) was Grade 3. No Grade 4 events or fatalities were observed (**Supplementary Table 1**).

**Table 4:** Characteristics of diarrhea−related adverse events.

Variable	n (%)
Patients (N=31)	
No diarrhea	2 (6.5%)
Any diarrhea	29 (93.5%)
Total number of diarrhea events in 29 patients (N=64)	
Treatment-emergent diarrhea events	
Grade 1	31 (48.4%)
Grade 2	32 (50.0%)
Grade 3	1 (1.6%)
Characteristics of grade ≥3 diarrhea events	
Episodes	1 (1.6%)
Duration of episode, days	10
Time to first episode, days	24
Action taken due to per diarrhea (any grade) ^†^	
Dose hold	19 (29.7%)
Dose reduction	7 (10.9%)
Discontinuation	19 (29.7%)
Hospitalization	0 (0.0%)
No action or missing	19 (29.7%)

^†^ Each episode of diarrhea was managed with a single documented primary intervention. Individual patients could experience multiple episodes, each addressed with distinct management actions; Dose hold, continuation of neratinib at the original daily dose despite the occurrence of diarrhea, as considered appropriate based on clinical assessment; Dose reduction, decrease in the daily dose of neratinib; Discontinuation: interruption of neratinib therapy, further categorized as temporary discontinuation (therapy is paused and subsequently resumed at the original dose following resolution of diarrhea) or permanent discontinuation (complete cessation of neratinib treatment).

Regarding management, dose hold was implemented in 19 events (29.7%), dose reduction in 7 events (10.9%), and treatment discontinuation (including both temporary and permanent discontinuations) in 19 events (29.7%). None of the events required hospitalization. Additionally, 19 events (29.7%) lacked documented management measures or had missing data.

Permanent treatment discontinuation attributable to diarrhea occurred in 5 patients (16.1%), including 4 of 17 patients (23.5%) in the dose-escalation group and 1 of 14 patients (7.1%) in the non–dose-escalation group.

### 3.4 EQ-5D-5L and FACIT-D scores over time

HRQoL was assessed using the EQ-5D-5L and FACIT-D scales. Valid score samples were collected at predetermined time points (Day 1, Day 30, Day 60, Day 90, Day 180, and Day 360), comprising 31, 31, 31, 26, 25, and 25 cases, respectively. The number of discontinuations at each corresponding time point was 0, 0, 0, 5, 6, and 6, respectively (**Table 5**).

**Table 5:** EQ-5D-5L and FACIT-D scores during follow-up.

HRQoL	D1	D30	D60	D90	D180	D360	p-Value(vs.D1)				
Valid,n	n=31	n=31	n=31	n=26	n=25	n=25	D30	D60	D90	D180	D360
Discontinued,n	n=0	n=0	n=0	n=5	n=6	n=6					
EQ-5D-5L Scores											
EQ VAS											
Mean(SD)	89.94(9.92)	84.16(12.64)	84.13(17.69)	86.50(10.22)	89.92(7.74)	92.84(7.19)	0.018	0.161	0.032	0.531	0.502
Median[Min,Max]	90.0[50.0,100.0]	89.0[50.0,100.0]	90.0[30.0,100.0]	90.0[60.0,100.0]	90.0[80.0,100.0]	95.0[70.0,100.0]					
Health Utility Index											
Mean(SD)	0.96(0.07)	0.89(0.15)	0.91(0.17)	0.94(0.08)	0.96(0.07)	0.97(0.06)	0.011	0.033	0.052	0.219	0.681
Median[Min,Max]	1.00[0.64,1.00]	0.95[0.36,1.00]	0.95[0.10,1.00]	0.95[0.65,1.00]	1.00[0.69,1.00]	0.78[0.64,1.00]					
FACIT-D Scores											
Mean(SD)	58.81(7.95)	66.90(11.39)	65.23(10.75)	62.31(10.44)	59.12(7.80)	59.12(10.58)	0.002	0.005	0.285	0.708	0.808
Median[Min,Max]	60.0[39.0,75.0]	68.0[40.0,85.0]	64.0[42.0,85.0]	59.5[45.0,84.0]	60.0[45.0,71.0]	61.0[17.0,74.0]					

EQ-5D-5L, EuroQoL 5-Dimensions 5-Levels; FACIT-D, Functional Assessment of Chronic Illness Therapy –Diarrhea; HRQoL, health-related quality of life.

#### 3.4.1 EQ-5D-5L Score

EQ VAS Score: At baseline, the mean patient self-assessment of overall health was 89.94 ± 9.92, indicating a favorable initial health status. This score significantly declined to 84.16 ± 12.64 at month 1 (D30) (p=0.018) and remained low at month 2 (D60) (84.13 ± 17.69, p=0.161), suggesting that perceived health had not recovered during this period. From month 3 (D90), the score began to steadily improve, reaching 86.50 ± 10.22 (p=0.032). By month 6 (D180), the score returned to baseline levels (89.92 ± 7.74, p=0.531) and further increased to 92.84 ± 7.19 at month 12 (D360) (p=0.502), indicating that long-term treatment resulted in a self-rated health status surpassing the initial baseline.

Health Utility Index: The baseline mean was 0.96 ± 0.07. Compared to baseline, the index significantly decreased to 0.89 ± 0.15 at Day 30 (p=0.011) and remained reduced at Day 60 (0.91 ± 0.17, p=0.033), reflecting a notable impact on patients’ health function during the early treatment phase. From Day 90 onward, the index demonstrated a steady recovery, reaching 0.94 ± 0.08 (p=0.052), approaching baseline levels but still exhibiting slight differences. By Day 180, the index fully returned to baseline (0.96 ± 0.07, p=0.219), and by Day 360, it slightly increased to 0.97 ± 0.06 (p=0.681), quantitatively confirming a progressive improvement in patients’ health status as treatment progressed (**Supplementary Figure 2**).

#### 3.4.2 FACIT-D score

The baseline mean score of this scale was 58.81 ± 7.95, indicating that the disease-related quality of life of patients was at a moderate to high level. Compared to baseline, the score significantly increased to 66.90 ± 11.39 at D30 (p=0.002) and remained elevated at D60 (65.23 ± 10.75, p=0.005). This transient improvement may be attributed to patients’ positive expectations regarding disease control during the early phase of treatment and their initial adaptation to symptom management strategies. Subsequently, the scores gradually declined, reaching 62.31 ± 10.44 at D90 (p=0.285), at which point the statistical difference from baseline was no longer observed. By D180 and D360, the scores stabilized at 59.12 (p=0.708 and p=0.808, respectively), fully returning to baseline levels. The overall trend demonstrates a pattern of “initial improvement followed by later stabilization,” with no evidence of long-term deterioration in quality of life.

## 4 Discussion

### 4.1 Clinical significance of changes in health-related quality of life

This study enrolled 31 patients diagnosed with early-stage HER2-positive breast cancer who underwent extended adjuvant therapy with neratinib following the completion of standard adjuvant anti-HER2 targeted treatment.

The primary objective of this study was to evaluate the applicability of the neratinib dose-escalation strategy in Chinese patients with early-stage HER2-positive breast cancer and to characterize the dynamic effects of treatment and diarrhea on HRQoL using patient-reported outcomes. Our findings demonstrate that the dose-escalation regimen is feasible in this population. The overall permanent discontinuation rate due to diarrhea was 16.1% (5/31), which is lower than the rates reported in trials without mandatory dose escalation (e.g., 17% in ExteNET trial (6)). However, the dose−escalation group exhibited a numerically higher discontinuation rate compared with the non−dose−escalation group (23.5% vs. 7.1%). This difference likely reflects the preferential inclusion of higher−risk patients in the dose−escalation strategy within a real−world, non−randomized context, rather than suggesting inherently poor tolerability of the escalation regimen itself.

**Figure 1:**
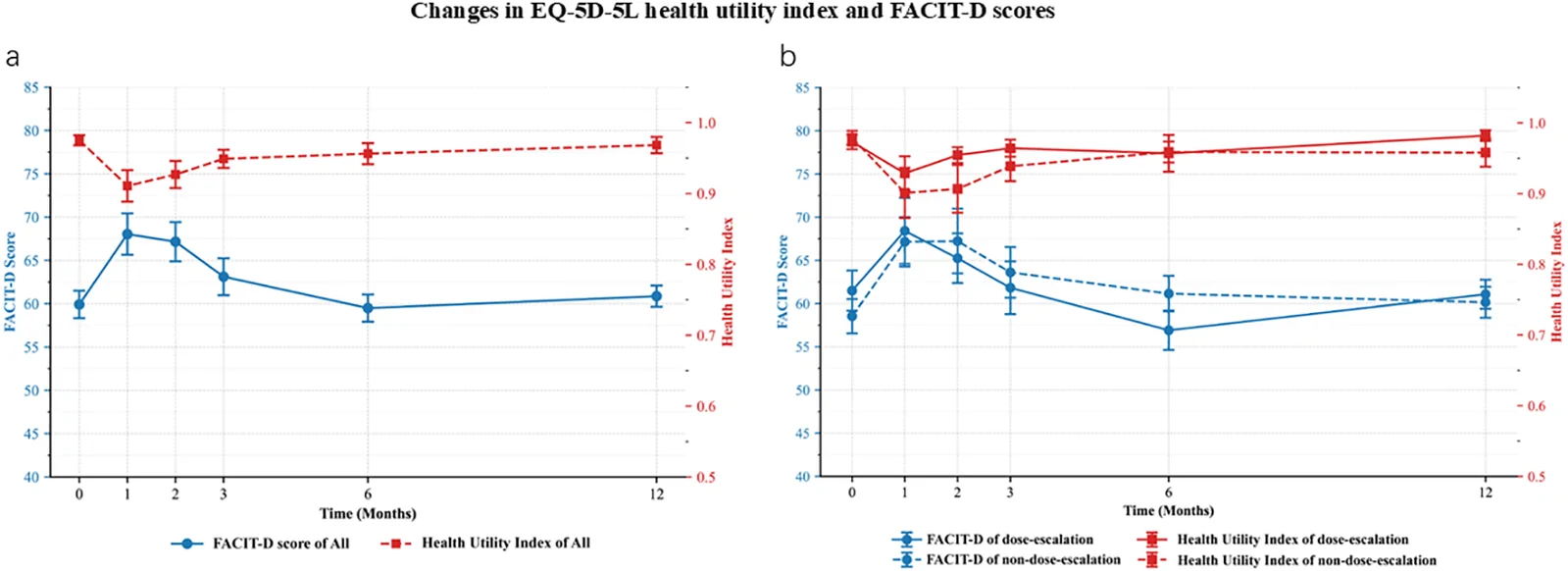
Analysis of changes in EQ-5D-5L health utility index and FACIT-D scores up to 360 days post-treatment discontinuation (valid response count, n=25).

At first glance, the higher rate of permanent treatment discontinuation due to diarrhea in the dose-escalation group (23.5%, 4/17) compared with the non-dose-escalation group (7.1%, 1/14) appears to contradict the rationale for dose escalation as a strategy to improve tolerability. However, several considerations are essential when interpreting this observation. First, as this was a non-randomized, real-world study, the assignment to dose-escalation versus full-dose initiation was determined by clinical judgment rather than random allocation. Second, despite the higher discontinuation rate, HRQoL outcomes did not differ significantly between groups at any time point (**Figure 1**), suggesting that the dose-escalation approach may have exerted a protective effect by mitigating the severity of diarrhea-related quality-of-life impairment among patients at elevated risk. Third, it is noteworthy that these patients experienced treatment-limiting diarrhea despite starting therapy at reduced doses, supporting the hypothesis that host-related factors, rather than initial dose intensity, were the predominant determinants of early discontinuation. Finally, the small subgroup sizes (n=17 and n=14) imply that the absolute number of events substantially influences the calculated percentages; thus, the difference of three patients between groups, although clinically relevant, should be interpreted with caution. These findings align with real-world evidence indicating that a subset of patients remains intolerant to neratinib even with dose escalation, highlighting the importance of individualized supportive care and the need for further investigation into predictive biomarkers of gastrointestinal toxicity.

**Figure 2:**
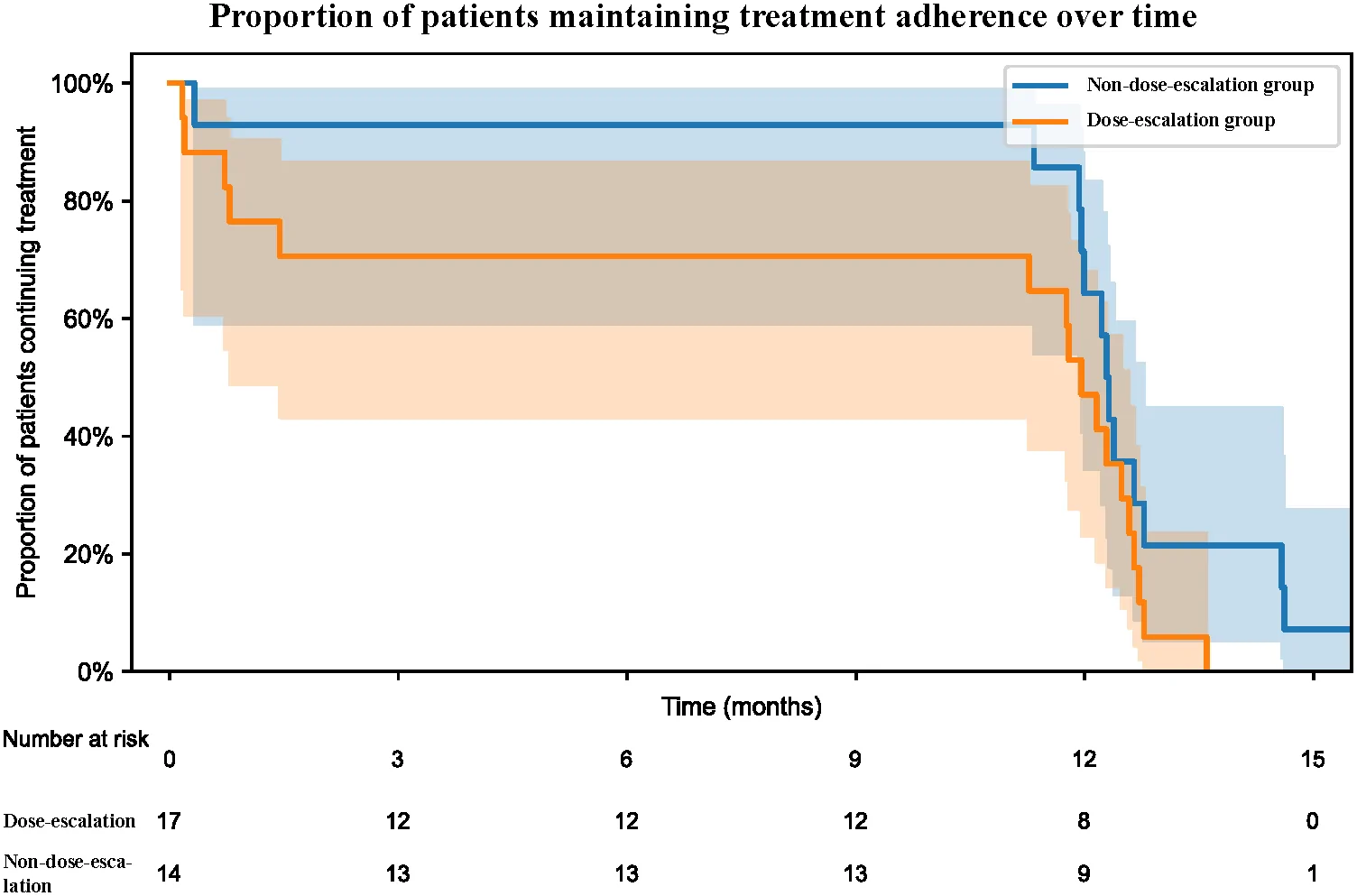
Treatment duration stratified by initial dose levels.

Notably, HRQoL trajectories were comparable between the dose-escalation and non-dose-escalation groups (**Figure 2**), indicating that the escalation strategy did not adversely affect quality of life despite a slightly higher incidence of early diarrhea. These results support the clinical utility of the dose-escalation approach as a standard practice to improve tolerability while maintaining treatment intensity.

HRQoL was longitudinally evaluated using the EQ-5D-5L and FACIT-D questionnaires at multiple time points: Day 1 (D1), Day 30 (D30), Day 60 (D60), Day 90 (D90), Day 180 (D180), and Day 360 (D360) of treatment. The findings revealed a temporary decline in HRQoL during the initial 30 to 60 days of therapy, which was subsequently followed by a progressive recovery, with HRQoL scores exceeding baseline levels between days 180 and 360. This pattern was consistently observed across key HRQoL metrics, including the EQ VAS, health utility index, and FACIT-D score, offering valuable patient-centered insights into the tolerability and impact of neratinib therapy.

The transient decline in HRQoL observed during the early treatment phase (Day 30–Day 60) is biologically plausible and closely linked to the established adverse event profile of neratinib. The EQ-5D-5L health utility index demonstrated a significant reduction from a baseline mean (SD) of 0.96 (0.07) to 0.89 (0.15) at Day 30 (P = 0.011), followed by partial recovery to 0.91 (0.17) at Day 60 (P = 0.681 compared to Day 1). Similarly, the EQ VAS score decreased from a baseline mean of 89.94 (9.92) to 84.16 (12.64) at Day 30 (P = 0.018). This early deterioration in HRQoL is predominantly attributed to treatment-related diarrhea, which was the most frequently reported adverse event in this study cohort. During the observation period, a total of 64 diarrhea events were recorded (**Table 4**). Among these, 31 (48.4%) were Grade 1, 32 (50.0%) were Grade 2, and 1 (1.6%) was Grade 3. Consistent with these findings, previous Phase III trials, such as the ExteNET trial (20), reported that over 90% of patients experienced diarrhea associated with neratinib treatment, with 21% developing Grade 3–4 diarrhea. Gastrointestinal toxicity not only directly compromises patients’ physical comfort but also contributes to fatigue, anxiety, and impaired daily functioning, collectively leading to the early decline in HRQoL observed in this study.

Notably, the recovery of HRQoL observed from Day 90 (health utility index: 0.94, standard deviation [SD] = 0.08; P = 0.033 compared to Day 1) and the sustained improvement at 12 months (health utility index: 0.97, SD = 0.06; EQ VAS: 92.84, SD = 7.19) represents a clinically significant finding. This recovery trajectory is closely linked to patients’ adaptation to neratinib and the efficacy of supportive care strategies. Additionally, dose modifications may have further enhanced treatment tolerability. This pattern of “early toxicity followed by subsequent HRQoL recovery” is consistent with findings from the NALA trial (21). The data demonstrate that PROs tend to stabilize within three months of initiating neratinib-based therapy, despite ongoing treatment. Importantly, this study extends these observations by confirming that HRQoL not only recovers but can surpass baseline levels after prolonged treatment. This finding underscores the absence of significant cumulative toxicity associated with neratinib and highlights the psychological relief experienced by patients upon completing effective adjuvant therapy.

### 4.2 Comparison with existing literature

The findings of this study align with prior research on neratinib and HRQoL in patients with breast cancer, while also offering significant supplementary insights. In the ExteNET trial, the efficacy of one-year intensified neratinib treatment was evaluated through PROs using the FACT-B and EQ-5D-3L scales. Consistent with the results of this study, the trial demonstrated a temporary decline in overall health status and physical functioning during the initial three months of treatment, followed by a recovery to baseline levels by the sixth month (22).

However, this study presents notable differences in several key aspects:

First, this study utilized the EQ-5D-5L scale, which provides a standardized health utility index—a critical metric for health economic evaluations and cross-study comparisons—whereas the ExteNET trial employed a disease-specific questionnaire. In this study, the health utility index at 12 months was 0.97 (SD = 0.06), which aligns with the overall health score of 75–80 on the EQ-5D-3L scale reported in the ExteNET trial at one year, thereby reinforcing the long-term tolerability of neratinib.

Second, this study specifically examined the context of intensified treatment following adjuvant therapy (after the completion of initial anti-HER2 treatment), whereas most prior studies included patients who initiated neratinib immediately after chemotherapy. The treatment completion rate in this study cohort was 80.6% (median treatment duration: 12.2 months), slightly exceeding the 73.1% reported in the ExteNET trial. This discrepancy may be attributed to improved patient selection, more comprehensive supportive care measures (e.g., routine loperamide prophylaxis), or reduced cumulative toxicity in the post-adjuvant treatment setting. Additionally, dose-dependent outcome analysis in this study revealed that patients initiating neratinib at the standard dose of 240 mg/day (comprising 45.2% of the cohort) demonstrated treatment persistence comparable to those in the lower-dose groups (120 mg/day or 160 mg/day), indicating that the standard dose is generally manageable for a substantial proportion of patients when appropriate supportive care is implemented. Moreover, these findings further substantiate current clinical practice guideline recommendations (23). However, the higher discontinuation rate observed in the dose−escalation group highlights the importance of individualized risk assessment and the need for further research to identify predictive factors associated with neratinib intolerance.

Third, this study utilized the FACIT-D scale, a validated instrument specifically designed to evaluate fatigue and quality of life in cancer patients. The findings demonstrated a significant increase in scores from a baseline of 58.81 (SD = 7.95) to 66.90 (SD = 11.39) on day 30 of treatment (P = 0.002) and 62.31 (SD = 10.44) on day 90 (P = 0.005), followed by stabilization. This early improvement in fatigue-related quality of life, though somewhat unexpected, may be attributed to psychological benefits associated with the initiation of targeted therapy. For patients with residual disease or high-risk features, the established efficacy of neratinib in reducing recurrence risk may offer considerable psychological reassurance. Notably, all patients underwent standardized pretreatment counseling and education regarding the therapeutic benefits and potential toxicities, which may have enhanced psychological reassurance and contributed to the early improvement observed in fatigue-related domains. The impact of such educational interventions on patient-reported outcomes warrants further investigation in prospective studies. Systematic reviews have previously highlighted that PROs are influenced by both physical symptoms and psychological factors, with perceived therapeutic benefits potentially alleviating the impact of mild-to-moderate adverse events (24).

In summary, the findings of this study underscore the importance of incorporating both physical and psychological dimensions in the evaluation of HRQoL during adjuvant therapy. This approach is essential to comprehensively capture patients’ treatment experiences and the associated changes in their quality of life.

### 4.3 Study contributions and limitations

The primary contribution of this study lies in the comprehensive longitudinal evaluation of HRQoL in patients with early-stage HER2-positive breast cancer undergoing neratinib intensification therapy. This assessment was conducted using two validated instruments, EQ-5D-5L and FACIT-D, within a real-world cohort (25–27). Unlike large phase III trials that predominantly focus on efficacy endpoints such as disease-free survival and often underrepresent vulnerable patient populations, this study provides detailed data on HRQoL changes over a 12-month period, offering valuable insights to inform clinical decision-making. In comparison to previous studies that primarily emphasize survival outcomes and toxicity profiles (28), this research uniquely delivers longitudinal quality-of-life data during the extended adjuvant treatment phase—a period that is rarely systematically evaluated in clinical trials (29). While prior systematic reviews have established neratinib’s efficacy in reducing recurrence risk, our findings further underscore that, despite an initial decline in HRQoL, most patients achieve and maintain acceptable quality of life over time. This supports the feasibility of long-term neratinib use in motivated patients receiving adequate supportive care. The observed long-term recovery and improvement in HRQoL address a critical unmet need, providing robust evidence that confirms the favorable long-term tolerability of neratinib, enabling patients and clinicians to balance the benefits of adjuvant therapy against its impact on quality of life.

Furthermore, the evaluation of treatment-related diarrhea and its management strategies, including loperamide prophylaxis (applied in a limited subset of patients, n=7) and dose modification, provides preliminary insights relevant to clinical practice. Notably, despite the high incidence of grade 2 diarrhea (50.0%), the hospitalization rate remained at 0%, indicating that proactive supportive care measures can effectively control this adverse event and mitigate its impact on HRQoL. However, due to the very limited number of patients who received prophylactic loperamide, definitive conclusions regarding its specific efficacy cannot be drawn, and exploratory subgroup analyses were not feasible. This finding is especially significant in resource-constrained healthcare settings and highlights the critical importance of implementing standardized protocols for diarrhea management.

It is noteworthy that, despite a reduction in the number of completed questionnaires during subsequent follow-ups, the study cohort demonstrated a relatively high retention rate in quality-of-life assessments. This observation may indicate a selective population with superior tolerability or adherence to the study protocol. Furthermore, the baseline characteristics of the patients were representative of a typical population diagnosed with early-stage HER2-positive breast cancer. However, the limited sample size posed constraints on conducting detailed subgroup analyses.

Despite the aforementioned strengths, several limitations of this study must be acknowledged. Firstly, the small sample size (n=31), coupled with the absence of a formal *a priori* sample size calculation, may have resulted in limited statistical power to detect subtle yet clinically meaningful differences. This limitation, along with potential attrition bias—given that only 25 patients completed the 12-month follow-up—may constrain the generalizability of the results. Additionally, because only a small number of patients received prophylactic loperamide (n=7), a comparative analysis of HRQoL outcomes based on prophylaxis status could not be conducted. Additionally, prophylactic loperamide was initiated only in a subset of patients within the dose−escalation group (n=7), while no patient in the non−dose−escalation group received prophylaxis at treatment initiation. This imbalance may confound comparisons of diarrhea severity and HRQoL between the two groups; however, the very limited number of prophylactic users likely minimizes its overall impact on the study findings. Future investigations employing standardized prophylactic protocols across all treatment groups are warranted to delineate the independent effect of prophylaxis from that of the dose−escalation strategy. Consequently, the specific impact of loperamide prophylaxis on the observed HRQoL trajectory remains undetermined in this study and warrants further investigation in future clinical trials. Nevertheless, the consistent trends in HRQoL changes observed across multiple time points and measurement scales partially address this concern. Moreover, considering that repeated assessments were performed at various time points and across multiple endpoints without adjustment for multiplicity, the potential inflation of type I error warrants careful consideration when interpreting the findings. Secondly, the single-center design may introduce selection bias, as the enrolled patients might possess better performance status and a higher willingness to undergo long-term treatment. Thirdly, patient-reported symptoms beyond diarrhea, such as nausea, vomiting, and rash, were not collected, despite their potential influence on HRQoL. Fourth, this study did not conduct multivariable analyses to control for potential confounding factors (e.g., age, performance status, baseline symptom burden), which may have influenced the observed HRQoL trajectories. Fifth, the exclusive reliance on patient-reported outcomes introduces the possibility of reporting bias, as responses may be affected by recall errors, social desirability, or the patient’s current health status at the time of assessment. Future large-scale, multicenter studies with extended follow-up periods are warranted to validate these findings and to further investigate the impact of other adverse events on HRQoL.

## 5 Conclusion

In summary, this study confirms that neratinib intensification therapy is associated with a transient decline in early HRQoL within the first 30–60 days, primarily attributable to treatment-related diarrhea. Subsequently, HRQoL gradually recovers and demonstrates sustained improvement over a 12-month period. The findings reveal an initial, temporary deterioration in patients’ quality of life, followed by stabilization and progressive recovery. This trajectory was consistently captured using both the generic EQ-5D-5L utility index and the cancer-specific FACIT-D scale, aligning with the established early toxicity profile of neratinib, which is predominantly driven by manageable gastrointestinal adverse events. These results are consistent with prior evidence but expand existing knowledge by providing detailed patient-reported outcomes in a real-world adjuvant treatment setting (30). Furthermore, the use of standardized scales facilitates cross-study comparisons, thereby enriching the current evidence base. The proven efficacy of neratinib in reducing recurrence risk, combined with the present study’s confirmation of its long-term tolerability, underscores its role as a standard treatment option for patients with early-stage HER2-positive breast cancer. Proactive supportive care measures—including loperamide prophylaxis and dose adjustments—are essential for mitigating adverse events and preserving HRQoL. Future research should prioritize the identification of patient-specific factors predictive of HRQoL changes to enable personalized treatment strategies and optimize clinical outcomes (31).

## Data Availability

The raw data supporting the conclusions of this article will be made available by the authors, without undue reservation.
