# Supplementary material for: Longitudinal evaluation of health-related quality of life in Chinese patients with early-stage HER2-positive breast cancer undergoing neratinib extended adjuvant therapy: a real-world prospective cohort study
Source: Front Oncol. 2026 Aug 12;16:1828599. doi: 10.3389/fonc.2026.1828599 (PMC13506311; doi:10.3389/fonc.2026.1828599)
Supplement: Supplementary file 1 [file DataSheet1.docx]

**Supplementary Figure 1.** Patient disposition.

**Supplementary Figure 2.** Changes in EQ-5D-5L Score (valid response count, N=25) .

**Supplementary Table 1.** Incidence and severity of diarrhea over time .

| Time point | Dose-escalation group (Total number of diarrhea events, N=26) | | | | Non-dose-escalation group (Total number of diarrhea events, N=38) | | | |
| --- | --- | --- | --- | --- | --- | --- | --- | --- |
|  | Any grade, n (%) | Grade 1, n (%) | Grade 2, n (%) | Grade ≥3, n (%) | Any grade, n (%) | Grade 1, n (%) | Grade 2, n (%) | Grade ≥3, n (%) |
| D30 | 22(84.5%) | 7(26.9%) | 14(53.8%) | 1(3.8%) | 21(55.3%) | 10(26.3%) | 11(29.0%) | 0 |
| D60 | 2(7.6%) | 1(3.8%) | 1(3.8%) | 0 | 5(13.1%) | 4(10.5%) | 1(2.6%) | 0 |
| D90 | 1(3.8%) | 1(3.8%) | 0 | 0 | 6(15.8%) | 3(7.9%) | 3(7.9%) | 0 |
| D180 | 0 | 0 | 0 | 0 | 4(10.5%) | 3(7.9%) | 1(2.6%) | 0 |
| D360 | 1(3.8%) | 1(3.8%) | 0 | 0 | 2(5.2%) | 1(2.6%) | 1(2.6%) | 0 |
